# Left ventricular wall findings in non-electrocardiography-gated contrast-enhanced computed tomography after extracorporeal cardiopulmonary resuscitation

**DOI:** 10.1186/s13054-019-2624-1

**Published:** 2019-11-14

**Authors:** Kazuhiro Sugiyama, Masamichi Takahashi, Kazuki Miyazaki, Takuto Ishida, Mioko Kobayashi, Yuichi Hamabe

**Affiliations:** 1 Tertiary Emergency Medical Center, Tokyo Metropolitan Bokutoh Hospital, 23-15 Kotobashi, 4-Chome, Sumida-ku, Tokyo, 130-8575 Japan; 2 The Department of Radiology, Tokyo Metropolitan Bokutoh Hospital, 23-15 Kotobashi, 4-Chome, Sumida-ku, Tokyo, 130-8575 Japan

**Keywords:** Extracorporeal cardiopulmonary resuscitation, Non-electrocardiography-gated computed tomography, Left ventricular wall, Hypoenhancement

## Abstract

**Background:**

Few studies have reported left ventricular wall findings in contrast-enhanced computed tomography (CE-CT) after extracorporeal cardiopulmonary resuscitation (ECPR). This study examined left ventricular wall CE-CT findings after ECPR and evaluated the association between these findings and the results of coronary angiography and prognosis.

**Methods:**

We evaluated out-of-hospital cardiac arrest patients who were treated with ECPR and subsequently underwent both non-electrocardiography-gated CE-CT and coronary angiography at our center between January 2011 and April 2018. Left ventricular wall CE-CT findings were classified as follows: (1) homogeneously enhanced (HE; the left ventricular wall was homogeneously enhanced), (2) segmental defect (SD; the left ventricular wall was not segmentally enhanced according to the coronary artery territory), (3) total defect (TD; the entire left ventricular wall was not enhanced), and (4) others. Successful weaning from extracorporeal membrane oxygenation, survival to hospital discharge, and predictive ability of significant stenosis on coronary angiography were compared among patients with HE, SD, and TD patterns.

**Results:**

A total of 74 patients (median age, 59 years) were eligible, 50 (68%) of whom had initial shockable rhythm. Twenty-three (31%) patients survived to hospital discharge. HE, SD, TD, and other patterns were observed in 19, 33, 11, and 11 patients, respectively. The rates of successful weaning from extracorporeal membrane oxygenation (84% vs. 39% vs. 9%, *p* < 0.01) and survival to hospital discharge (47% vs. 27% vs. 0%, *p* = 0.02) were significantly different among patients with HE, SD, and TD patterns. In post hoc analysis, patients with HE patterns had a significantly higher success rate of weaning from extracorporeal membrane oxygenation than those with SD and TD patterns. SD predicted significant stenosis with a sensitivity of 74% and specificity of 94%.

**Conclusions:**

Homogenously enhanced left ventricular wall might be a predictor of good left ventricular function recovery. In contrast, total enhancement defect in the entire left ventricular wall was associated with poor outcomes. Contrast defect matching the coronary artery territory could predict significant coronary artery stenosis with good specificity. The left ventricular wall findings in non-electrocardiography-gated CE-CT after ECPR might be useful for diagnosis and prognostic prediction.

## Background

Extracorporeal cardiopulmonary resuscitation (ECPR) is a promising treatment for refractory out-of-hospital cardiac arrest (OHCA) [1–3]. Computed tomography (CT) is often performed after ECPR for the determination of etiology and evaluation of complications. Although coronary artery disease is a leading cause of refractory ventricular fibrillation in OHCA patients [4, 5], the findings from enhanced CT of the left ventricular wall have not been adequately considered. Hypoenhancement of the left ventricular wall in the early phase on electrocardiography (ECG)-gated contrast-enhanced CT (CE-CT) has been reported to be associated with significant stenosis of the coronary artery and diagnosis of acute myocardial infarction in patients with acute chest pain [6]. Although ECG gating is essential for evaluating the coronary artery in CE-CT, this method requires a special technique. Non-ECG-gated CE-CT is a common modality for evaluating emergency patients. Even in non-ECG-gated CE-CT performed before coronary angiography (CAG), early defects of the left ventricular wall can predict non-ST segment elevation myocardial infarction with good sensitivity and specificity [7].

Few studies have reported left ventricular wall findings on non-ECG-gated CE-CT after ECPR. In this study, we evaluated the association between these findings and the results of CAG and prognosis.

## Methods

### Patients

This retrospective study included OHCA patients who were treated with ECPR and underwent both non-ECG-gated CE-CT and CAG at the tertiary emergency care center of Tokyo Metropolitan Bokutoh Hospital between January 2011 and April 2018. In this study, patients who underwent CAG before CE-CT were excluded. The baseline demographic and clinical characteristics of the patients were collected from their medical records, and the timing of pre-hospital events was recorded according to the reports of emergency medical service personnel. The institutional review board of Tokyo Metropolitan Bokutoh Hospital approved the study (institutional approval reference number 30-056), which complied with the tenets of the Declaration of Helsinki. The requirement for the acquisition of informed consent from patients was waived owing to the retrospective design of the study.

### Protocol for ECPR and post-cardiac arrest care

The indications for ECPR at our institution are as follows: (i) OHCA patients aged ≤ 65 years with initial shockable rhythm and witness and (ii) OHCA patients aged ≤ 70 years with presumed reversible etiology who collapsed after the arrival of emergency medical service personnel. In this case, any initial rhythm was acceptable. Patients with very long transfer times and terminal illnesses were excluded. The implementation of ECPR is decided at the discretion of the individual emergency physician. Therefore, some cases did not meet the rigid indications.

ECPR was implemented immediately after the patient’s arrival at the emergency room. In all cases, we selected the ipsilateral or contralateral femoral vein and artery for cannulation of venous and arterial cannulas. Size 16-French (Fr) cannulas were chosen for the femoral artery, and size 22-Fr cannulas were chosen for the femoral vein. Cannulation was performed percutaneously with the Seldinger technique under an ultrasonic guide. When our emergency room was renovated in August 2014, an interventional radiology-CT system was installed [8]. Prior to this (until July 2014), a cannula was placed under ultrasonic guidance only, and after August 2014, cannulas were placed under both ultrasonic and fluoroscopic guidance. The extracorporeal membrane oxygenation (ECMO) circuit, including a centrifugal pump, a hollow fiber oxygenator (MERA CPB circuit; Senko Medical Instrument Mfg. Co. Ltd., Tokyo, Japan or Capiox EBS; Terumo Corporation, Tokyo, Japan), and a heparin-coated surface circuit, was primed using normal saline with 3000 units of heparin. After venous and arterial cannulas were successfully inserted, the ECMO circuit was connected and the ECMO pump flow was set at 4 L/min at the start. After the start of ECMO, a 4-Fr sheath was placed in the superficial femoral artery to prevent distal limb ischemia.

All patients were resuscitated according to the current recommendations [9, 10]. The patients received appropriate amounts of fluid or vasopressors to maintain a mean blood pressure above 65 mmHg and were ventilated to maintain normocarbia and prevent hypoxia and hyperoxia (PaO_2_ > 300 mmHg). Patients in whom cardiac etiology was suspected underwent CAG and percutaneous coronary intervention (PCI), if indicated. Patients who remained comatose after the initiation of ECMO were treated with targeted temperature management at 34 °C for 24 h and subsequently rewarmed to 36 °C for the next 12 h using a heat exchanger in the circuit.

Neurological outcomes were predicted based on the results of clinical examinations performed at least 72 h after return of spontaneous circulation (ROSC) and of brain CT. Neurological outcomes were predicted as poor when (1) a patient remained unconscious for at least 72 h post-ROSC with a Glasgow Coma Scale motor response score of ≤ 2, (2) there was no pupillary reflex, and (3) diffuse anoxic injury was recognized post-ROSC or during follow-up brain CT (on days 4–5) [9]. Even in these patients, we did not withdraw treatment, and ongoing life-sustaining measures were continued. However, additional aggressive treatment modalities, such as hemodialysis and additional mechanical circulatory support devices, were withheld.

### CT protocol

After the initiation of ECMO, plain head CT and non-ECG-gated CE-CT of the whole body were performed before CAG in all eligible patients. Non-ECG-gated CE-CT was performed using a 64-slice CT scanner (Aquilion CX TSX-101A®; Cannon Medical Systems Corp., Tochigi, Japan), with the following settings: slice thickness, 0.5 mm; tube voltage, 120 kV; tube current, 350–500 mA (based on patient size); and gantry rotation time, 500 ms. Contrast medium (2 mL/kg [maximum 100 mL] of iopamidol [370 mg iodine/mL]) was intravenously injected from the peripheral vein of the left or right arm at a rate of 3.0–3.5 mL/s. Scans were performed at 30 s and 90 s after contrast injection.

### Classification of left ventricular wall findings on non-ECG-gated CE-CT

The left ventricular walls were evaluated using the original axial images, reformatted short-axis images, and horizontal and vertical long-axis images scanned at 90 s after contrast injection.

Before evaluating the eligible patients, we set a reference CT value range for the well-enhanced left ventricular wall. We examined each CT value of the standard 17 segments of the left ventricle [11] of 20 previously healthy patients aged between 20 and 40 years. They presented at our center after blunt trauma, showed stable hemodynamics, underwent non-ECG-gated CE-CT on admission, and revealed no cardiac problems during hospitalization. The 99th percentile of these CT values was set as a referenced normal range of a well-enhanced left ventricular wall, and this range was 80–167 HU. A color map of the left ventricular wall was created for each CT image. The well-enhanced region with CT values within the normal range was displayed as light green, the non-enhanced or hypoenhanced region below the normal range as red, and the region above this normal range as light yellow. These images were created using Ziostation (Ziosoft Inc., Tokyo, Japan).

**Fig. 1:**
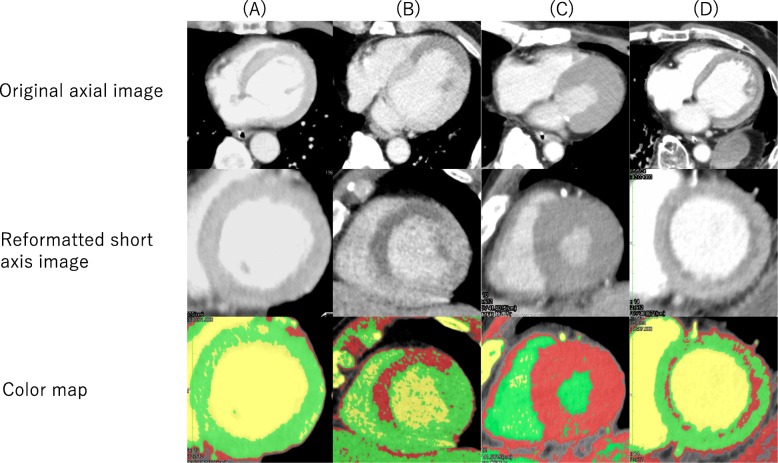
Classification of left ventricular wall patterns on non-ECG-gated CE-CT. **a** Homogeneously enhanced (HE): the left ventricular wall is homogeneously enhanced. **b** Segmental defect (SD): the left ventricular wall is not segmentally enhanced according to coronary artery territory. **c** Total defect (TD): more than 75% of the wall in the entire left ventricle is not enhanced. **d** Others: in this patient, the enhancement defect is limited to the subendomyocardial wall in the entire left ventricle. On the color map, the well-enhanced region within the normal range of CT values is displayed as light green, the non-enhanced or hypoenhanced region below the normal range as red, and the region above the normal range as light yellow

We classified left ventricular wall findings into four patterns (Fig. 1). The first pattern was homogeneously enhanced (HE), in which the entire left ventricular wall was homogeneously enhanced. The second was segmental defect (SD), in which the left ventricular wall was not segmentally enhanced according to the coronary artery territory. This pattern included both subendomyocardial and transmural hypoenhancement. The third was total defect (TD), in which more than 75% of wall thickness was not enhanced in the entire left ventricle. The fourth pattern included all other findings. These images were evaluated by a board-certified radiologist who was blinded to the results.

### Outcomes

Weaning from ECMO was defined as successful if the patient survived more than 48 h after the removal of cannulas of ECMO. This was the primary outcome. The secondary outcomes were survival to hospital discharge and predictive ability of significant stenosis on CAG, which were compared among patients with HE, SD, and TD patterns. In this study, ≥ 75% stenosis of the coronary artery branch, including the left main trunk, was defined as significant.

### Statistical analysis

Continuous variables were reported as medians with interquartile ranges (IQRs), and dichotomous variables were reported as numbers with percentages. The chi-square test for categorical variables and the Kruskal–Wallis test for continuous variables were used to compare the differences in patient characteristics for each pattern. Among patients with HE, SD, and TD patterns, differences in the rates of successful weaning from ECMO and survival to hospital discharge were examined using chi-square test. Bonferroni test was performed as post hoc test to examine the difference among patterns. Furthermore, the trend for the rates of successful weaning from ECMO and survival to hospital discharge was evaluated in the order of HE, SD, and TD patterns using the Cochran–Armitage test for trend. All statistical analyses were performed using EZR (Saitama Medical Center, Jichi Medical University, Saitama, Japan) [12].

## Results

**Fig. 2:**
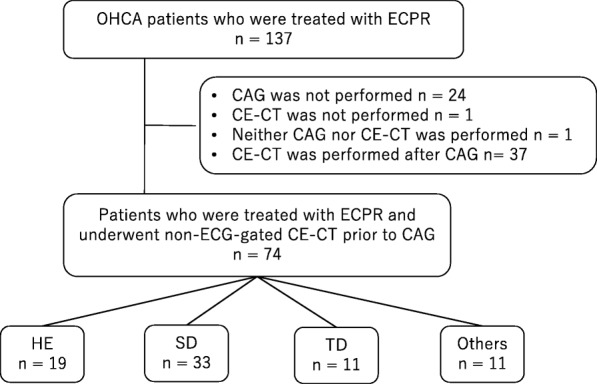
Flow chart of patient selection. OHCA, out-of-hospital cardiac arrest; ECPR, extracorporeal cardiopulmonary resuscitation; CAG, coronary angiography; CE-CT, contrast-enhanced computed tomography; ECG, electrocardiography; HE, homogeneously enhanced; SD, segmental defect; TD, total defect

During the study period, a total of 137 OHCA patients were treated with ECPR at our center. Of these patients, 26 patients did not undergo CAG or CE-CT. CAG was performed prior to CE-CT in 37 patients. Therefore, a total of 74 patients were eligible for this study. Among these patients, 19 patients showed the HE pattern, 33 showed the SD pattern, 11 showed the TD pattern, and 11 showed “other pattern” (Fig. 2).

The characteristics of all patients according to the different patterns are shown in Table 1. Of the 74 patients in total, the median age of patients was 59 (IQR, 48–65) years, with 65 (88%) male patients and 65 (88%) patients who had a witnessed collapse. Only 40 (54%) patients underwent bystander CPR. Fifty (68%) patients had an initial shockable rhythm. The median time from collapse to ECMO was 45 (IQR, 40–55) min. The median lactate level at hospital arrival was 14.0 (IQR, 10.7–16.0) mmol/L. No significant differences in these variables based on the type of pattern were observed. Of all 74 patients, 43 (58%) had acute coronary syndrome. Twenty-three (31%) patients had other cardiac etiologies, which included cardiomyopathy, valvular heart disease, Wolff–Parkinson–White syndrome, and idiopathic ventricular fibrillation. Eight (11%) patients had non-cardiac etiologies. Acute coronary syndrome was the major etiology among patients with SD pattern, whereas other cardiac etiologies and non-cardiac etiologies were the major etiologies among patients with HE pattern.

**Table 1:** Patient characteristics in each pattern

	All patients, *n* = 74	HE, *n* = 19	SD, *n* = 33	TD, *n* = 11	Other, *n* = 11	*p* value
Age (years)*	59 (48–65)	54 (37–62)	59 (53–64)	54 (50–63)	62 (49–69)	0.23
Male, *n* (%)	65 (88%)	15 (79%)	30 (91%)	11 (100%)	9 (82%)	0.31
Witnessed collapse, *n* (%)	65 (88%)	17 (90%)	27 (82%)	11 (100%)	10 (91%)	0.43
Bystander CPR, *n* (%)	40 (54%)	8 (42%)	20 (61%)	6 (55%)	6 (55%)	0.65
Initial shockable rhythm, *n* (%)	50 (68%)	15 (79%)	18 (55%)	9 (82%)	8 (73%)	0.51
Time from collapse to initiation of ECMO flow (min)*	45 (40–55)	54 (49–61)	44 (36–53)	45 (42–46)	45 (38–66)	0.37
Lactate level at hospital arrival (mmol/L)*	14.0 (10.7–16.0)	14.0 (10.9–16.0)	12.6 (8.9–16.0)	14.0 (10.8–16.0)	14.2 (11.6–15.8)	0.89
Etiology of cardiac arrest						< 0.01
Acute coronary syndrome, *n* (%)	43 (58%)	1 (5%)	31 (94%)	6 (55%)	5 (45%)	
Other cardiac etiologies, *n* (%)	23 (31%)	12 (63%)	2 (6%)	4 (36%)	5 (45%)	
Non-cardiac etiologies, *n* (%)	8 (11%)	6 (32%)	0	1 (9%)	1 (9%)	

*HE* homogeneously enhanced, *SD* segmental defect, *TD* total defect, *CPR* cardiopulmonary resuscitation, *ECMO* extracorporeal membrane oxygenation

*Median (interquartile range)

**Fig. 3:**
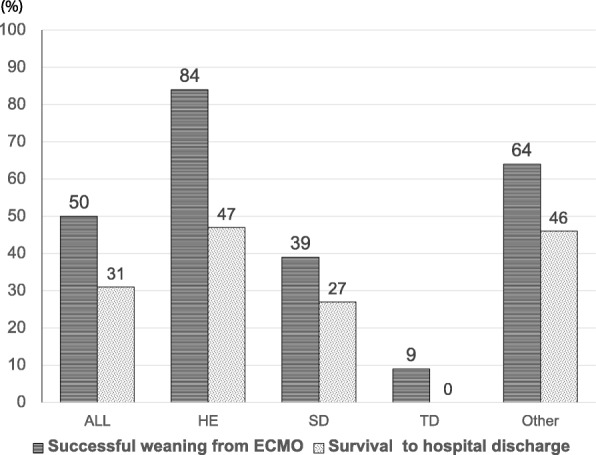
Successful weaning from ECMO and survival to hospital discharge according to each left ventricular wall pattern. ECMO, extracorporeal membrane oxygenation; HE, homogeneously enhanced; SD, segmental defect; TD, total defect

Among all patients, 37 (50%) were successfully weaned from ECMO and 23 patients (31%) survived. Among patients with HE pattern, 16 (84%) were weaned from ECMO and nine (47%) survived. Among patients with SD pattern, 13 (39%) were weaned from ECMO and nine (27%) survived. Among patients with TD pattern, only one (9%) was weaned from ECMO and this patient did not survive. The rates of successful weaning from ECMO (84% vs. 39% vs. 9%, *p* < 0.01) and survival to hospital discharge (47% vs. 27% vs. 0%, *p* = 0.02) were significantly different among patients with HE, SD, and TD patterns. Post hoc analysis showed that patients with HE pattern had a significantly higher rate of successful weaning from ECMO than those with SD (*p* = 0.01) and TD patterns (*p* < 0.01). Both the rates of successful weaning from ECMO (*p* = 0.01) and survival to hospital discharge (*p* = 0.01) showed a significant decreasing trend in the order stated above (i.e., HE, SD, and TD) (Fig. 3).

Of all 74 patients, 42 (57%) had significant coronary artery stenosis on CAG. Among these patients, 17 had one-vessel disease, 19 had two-vessel disease, and 6 had three-vessel disease. Two out of 19 patients (11%) with HE pattern, 31 out of 33 patients (94%) with SD pattern, and five out of 11 patients (45%) with TD pattern had significant coronary artery stenosis (Table 2). The sensitivity of the SD pattern for predicting significant stenosis on CAG was 74% (95% CI, 58–86%), the specificity was 94% (79–99%), the positive predictive value (PPV) was 94% (80–99%), and the negative predictive value (NPV) was 73% (57–86%). The area under the receiver operating characteristic curve (AUROC) was 0.84 (95% CI, 0.76–0.92). The sensitivity, specificity, PPV, NPV, and AUROC were low in the TD pattern and “other pattern.” On the contrary, the sensitivity of the HE pattern for predicting the absence of significant stenosis of the coronary artery was 53% (35–71%), the specificity was 95% (84–99%), the PPV was 90% (67–99%), the NPV was 73% (59–84%), and the AUROC was 0.74 (95% CI, 0.65–0.84) (Table 3). Two out of 19 patients (11%) with HE pattern, 11 out of 33 patients (33%) with SD pattern, one out of 11 patients (9%) with TD pattern, and one patient (9%) with “other pattern” revealed ST segment elevation on ECG before non-ECG-gated CE-CT.

**Table 2:** Significant stenosis on coronary angiography in each pattern

	All patients. *n* = 74	HE, *n* = 19	SD, *n* = 33	TD, *n* = 11	Other, *n* = 11
Significant stenosis of the coronary artery, *n* (%)	42 (57%)	2 (11%)	31 (94%)	5 (45%)	4 (36%)
One-vessel disease, *n* (%)	17 (23%)	2 (11%)	13 (39%)	1 (9%)	1 (9%)
Two-vessel disease, *n* (%)	19 (26%)	0	14 (42%)	3 (27%)	2 (18%)
Three-vessel disease, *n* (%)	6 (8%)	0	4 (12%)	1 (9%)	1 (9%)
Spasm of the coronary artery, *n* (%)	2 (3%)	0	0	1 (9%)	1 (9%)

*HE* homogeneously enhanced, *SD* segmental defect, *TD* total defect

**Table 3:** Sensitivity, specificity, PPV, NPV, and AUROC of each pattern for predicting the result of coronary angiography

	Sensitivity (95% CI)	Specificity (95% CI)	PPV (95% CI)	NPV (95% CI)	AUROC (95% CI)
Prediction of the presence of significant stenosis					
SD	74 (58–86)	94 (79–99)	94 (80–99)	73 (57–86)	0.84 (0.76–0.92)
TD	12 (4–26)	81 (64–93)	46 (17–77)	41 (29–54)	0.47 (0.38–0.55)
Other	10 (3–23)	78 (60–91)	36 (11–69)	40 (28–53)	0.44 (0.35–0.52)
Prediction of the absence of significant stenosis					
HE	53 (35–71)	95 (84–99)	90 (67–99)	73 (59–84)	0.74 (0.65–0.84)

*PPV* positive predictive value, *NPV* negative predictive value, *AUROC* area under the receiver operating characteristic curve, *HE* homogenously enhanced, *SD* segmental defect, *TD* total defect, *CI* confidence interval

Among patients with SD pattern, the median number of hypoenhanced segments out of the standard 17 segments [11] was 9 (IQR, 6–11). Fifteen patients with eight or fewer hypoenhanced segments had a higher rate of successful weaning from ECMO than 18 patients with 9 or more hypoenhanced segments (60% vs. 22%, *p* = 0.04). The rate of survival to hospital discharge was not significantly different (40% vs. 17%, *p* = 0.23).

## Discussion

In the present study, we focused on left ventricular wall findings on non-ECG-gated CE-CT after ECPR in OHCA patients. We classified these findings into four separate patterns and showed that left ventricular wall finding patterns might be associated with prognosis and might be helpful in predicting significant stenosis of the coronary arteries. Patients with HE pattern had a higher success rate of weaning from ECMO and showed a trend toward higher survival than those with SD and TD patterns. Conversely, the TD pattern was associated with poor outcomes, and the SD pattern could predict coronary artery stenosis with good specificity. To the best of our knowledge, this is the first report on left ventricular wall findings on non-ECG-gated CE-CT after ECPR.

Each pattern used in this study had its own unique characteristics associated with diagnosis and prognosis, and this might be useful in customizing post-cardiac arrest care. The HE pattern might be an appropriate predictor of good left ventricular functional recovery after ECPR. Furthermore, the HE pattern had relatively high specificity, PPV, and AUROC for predicting the absence of significant stenosis of the coronary arteries. However, a few patients with this pattern show significant stenosis, and the need for CAG cannot be completely excluded. It has been reported that patients with unstable angina or recanalization of an occluded lesion may show the HE pattern [7]. In this study, two patients with HE pattern had significant coronary artery stenosis, and one patient had cardiac arrest due to cardiogenic pulmonary edema, with CAG showing 90% stenosis of the left anterior descending artery with abundant collateral flow. Another patient showed 75% stenosis of the left anterior descending artery, and the culprit lesion with TIMI grade 3 flow had already been recanalized. The patient subsequently underwent PCI because the plaque of the culprit lesion was unstable.

The SD pattern predicted significant coronary artery stenosis with good specificity (Additional file 1: Figure S1). In this study, we evaluated patients who underwent CE-CT before CAG. The best timing for CE-CT in patients treated with ECPR remains controversial. Nonetheless, we could avoid the risk of performing CAG or PCI in patients with contraindication to these procedures by using a CT-first approach. When CT has been performed, CAG should be highly recommended for patients with SD pattern. Furthermore, in patients with stenosis of multiple coronary arteries, contrast agents may help identify the defect and determine the true culprit of the index event. Regions of old infarction may also show as an enhancement defect. Potentially, the old infarction could be distinguished from the acute infarction based on wall thinning and the presence of intramyocardial fat with lower CT values [7, 13]. We only classified patients into the SD pattern group when the enhancement defect was matched with the coronary artery territory referencing the standardized myocardial segmentation [11]. This could lead to lower false-positive rates for predicting significant stenosis, avoiding the artifact caused by motion or beam hardening, and the degenerative changes in the heart [14].

The TD pattern is a unique finding in patients treated with peripheral VA-ECMO, and it was associated with poor outcomes in this study. Of all patients with TD pattern, five patients did not recover their own heartbeat and six patients showed very weak contraction after the initiation of ECMO. Some patients showed extremely slow flow on CAG even in the non-occluded coronary artery. If cardiac function is absent or very weak, the retrograde flow from the arterial cannula increases the end-diastolic pressure of the left ventricle [15]. The extent of early left ventricular wall enhancement is correlated with the coronary blood flow of each segment, and coronary perfusion pressure (aortic diastolic pressure minus left ventricular end-diastolic pressure) is one of the major determinants of coronary blood flow [16, 17]. In patients with TD pattern, coronary perfusion pressure might be very low because of the relatively low aortic diastolic pressure and elevated left ventricular pressure. This might result in the diffuse hypoenhancement of the entire left ventricle. Therefore, the TD pattern might suggest the need for venting of the left ventricle. Furthermore, the switch to a more durable mechanical circulatory device should be considered in these patients if a good neurological prognosis is expected, as the TD pattern may predict poor left ventricular functional recovery.

ECG-gated CE-CT is generally performed to evaluate coronary artery and left ventricular findings to obtain a better image by minimizing the motion artifact [18] and has been employed in most studies evaluating the effectiveness of CE-CT in diagnosing acute coronary syndrome [6, 19, 20]. Only a few studies have reported the effectiveness of non-ECG-gated CE-CT [7, 14, 21]. In this study, all images of the left ventricle scanned by non-ECG-gated CE-CT were acceptable for the evaluation of the patterns used in the present study. Consequently, the left ventricular findings were able to provide us with important information, on par with the information that we can obtain with other body parts, even in non-ECG-gated CE-CT after ECPR.

Enhanced CT images are unique in patients treated with peripheral veno-arterial ECMO. The degree of the enhancement of the left atrium, left ventricle, and ascending aorta is determined by complicated factors, including the timing of the scan, the place of the tip of the drainage cannula, the root of the injection of the contrast agents, and the mixing point of the flow from the patient’s heart and the retrograde flow from the arterial cannula placed via the femoral artery. In some cases, the ascending aorta is not homogeneously enhanced in the early phase after the injection of contrast agents because of the variation in these factors. To evaluate left ventricular wall enhancement, the Valsalva sinus needs to be homogenously enhanced and the contrast agents have to flow into the coronary arteries. In this study, Valsalva sinus enhancement was not homogeneous in images scanned at 30 s after the injection of contrast agents in several patients; however, the Valsalva sinus was homogenously enhanced at 90 s in most patients. Therefore, we evaluated the left ventricular wall using the images scanned at 90 s.

This study had some important limitations. First, this was a retrospective single-center study with a small sample size. Second, inter-observer differences should be considered in the evaluation of left ventricular wall findings on non-ECG-gated CE-CT. In this study, one board-certified radiologist who was blinded to the results evaluated these findings. Although inter-observer differences are reported as acceptable for the evaluation of myocardial hypoenhancement in patients with acute chest pain [20], further studies are required to validate the results of this study. Third, this study only included patients who underwent CE-CT before CAG. In patients who underwent CE-CT after CAG, the contrast agents used in CAG might have remained in the left ventricular wall during CE-CT. These findings are reported to be associated with poor recovery of the function of the affected segment [22]. These findings were not evaluated in this study and are worth investigating in future studies. Fourth, we simply classified left ventricular wall findings into HE, SD, and TD patterns. However, 11 patients could not be classified into these patterns and were categorized as having “other pattern.” These patients showed diffuse subendomyocardial hypoenhancement in the entire left ventricle or patchy hypoenhancement in the left ventricular wall and accounted for 15% of all eligible patients. Furthermore, “other pattern” had little value in predicting prognosis and stenosis of the coronary artery. This is also an important limitation of this study.

## Conclusions

Patients with HE pattern had a higher success rate of weaning from ECMO and showed a trend toward higher survival than those with SD and TD patterns. Conversely, the TD pattern was associated with poor outcomes. The SD pattern could predict coronary artery stenosis with good specificity. The left ventricular wall findings on non-ECG-gated CE-CT after ECPR might be useful for diagnoses and prognostic predictions. However, this was a pilot study with a limited sample size, and the findings need to be validated in further studies.

## Supplementary information

**Additional file 1 : Figure S1.** Findings of segmental defect (SD) on non-ECG-gated CE-CT in a patient with 90% stenosis of the left main trunk.(A) Usual axial image. (B) Reformatted short axis image. (C) Color map of reformatted short axis image. (D) Reformatted long axis image. (E) Intact right coronary artery on coronary angiography. (F) 90% stenosis of the left main trunk on coronary angiography.

## Data Availability

The datasets used and/or analyzed during the current study are available from the corresponding author on reasonable request.

## Abbreviations

ECPR: Extracorporeal cardiopulmonary resuscitation
CT: Computed tomography
CE-CT: Contrast-enhanced computed tomography
CAG: Coronary angiography
HE: Homogeneously enhanced
SD: Segmental defect
TD: Total defect
ECMO: Extracorporeal membrane oxygenation
OHCA: Out-of-hospital cardiac arrest
ECG: Electrocardiography
ROSC: Return of spontaneous circulation
PPV: Positive predictive value
NPV: Negative predictive value
AUROC: Area under the receiver operating characteristic curve
PCI: Percutaneous coronary intervention
