# Supplementary figures and images for: Left ventricular wall findings in non-electrocardiography-gated contrast-enhanced computed tomography after extracorporeal cardiopulmonary resuscitation
Source: Crit Care. 2019 Nov 14;23:357. doi: 10.1186/s13054-019-2624-1 (PMC6854640; doi:10.1186/s13054-019-2624-1)

## Slide 1
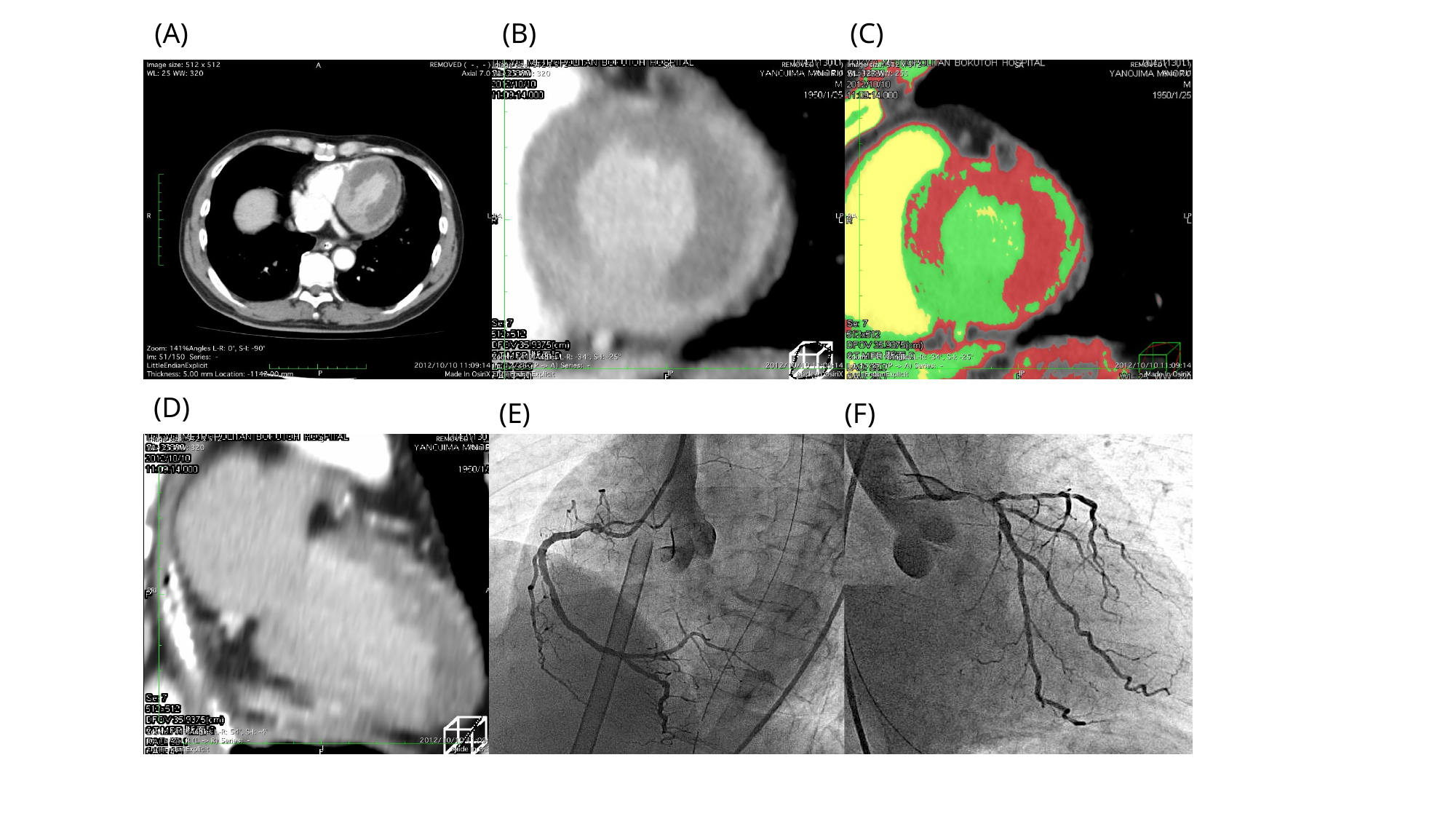

(A)
(B)
(C)
(D)
(F)
(E)

Supplement: Supplementary file 1 — Additional file 1 : Figure S1. Findings of segmental defect (SD) on non-ECG-gated CE-CT in a patient with 90% stenosis of the left main trunk.(A) Usual axial image. (B) Reformatted short axis image. (C) Color map of reformatted short axis image. (D) Reformatted long axis image. (E) Intact right coronary artery on coronary angiography. (F) 90% stenosis of the left main trunk on coronary angiography. [file 13054_2019_2624_MOESM1_ESM.pptx]
